# Therapeutic effects of different doses of prebiotic (isolated from S*accharomyces cerevisiae*) in comparison to n-3 supplement on glycemic control, lipid profiles and immunological response in diabetic rats

**DOI:** 10.1186/s13098-020-00576-6

**Published:** 2020-08-10

**Authors:** Janina de Sales Guilarducci, Breno Augusto Ribeiro Marcelino, Isaac Filipe Moreira Konig, Tamira Maria Orlando, Mary Suzan Varaschin, Luciano José Pereira

**Affiliations:** 1 Departamento de Ciências da Saúde – DSA, Universidade Federal de Lavras – UFLA, 3037, Lavras, 37200-000 Brazil; 2 Departamente de Medicina Veterinária – DMV, Universidade Federal de Lavras – UFLA, 3037, Lavras, 37200-000 Brazil

**Keywords:** Dietary fibers, Beta-glucans, Prebiotics, Metabolism

## Abstract

**Background:**

The regular intake of fiber generates numerous health benefits. However, the efficacy depends on the duration of consumption and the ingested dose. Studies investigating the optimal dose are of interest to enable the inclusion of fiber in the routine treatment of diabetic patients.

**Objective:**

We aimed to evaluate the effects of different doses of β-glucan (BG—isolated from *Saccharomyces cerevisiae*), in comparison to n-3 supplement, on the inflammatory and metabolic parameters of *Wistar* rats induced to diabetes by streptozotocin.

**Methods:**

Forty animals were randomly divided into six groups receiving 0 mg/kg, 10 mg/kg, 20 mg/kg, or 40 mg/kg BG daily for 4 weeks or fish oil derivative [1000 mg/kg of omega-3 fatty acids (n-3)] for the same period. One additional group was composed of healthy controls. Serum metabolic and immunological parameters were evaluated by colorimetric and ELISA assays respectively. Histopathological analysis of the liver, small intestine and pancreas were also conducted. Significant changes due to BG intake were set into regression models with second-degree fit in order to estimate the optimal BG dose to achieve health benefits.

**Results:**

The animals that ingested BG had lower food and water intake (p < 0.05) than the negative control group (0 mg/kg). However, consumption was still elevated in comparison to healthy controls. Blood glucose and serum levels of total cholesterol, LDL-c, and TG (p < 0.05) reduced in comparison to diabetic animals without treatment (better or similar to n-3 group depending on dose), but did not reach normal levels (in comparison to healthy controls). HDL-c was not different (p > 0.05) among all groups. These reductions were already seen with the lowest dose of 10 mg/kg. On average, the serum levels of the hepatic enzymes ALT and AST were 40% and 60% lower in the BG groups in comparison to diabetic animals without treatment (better results than n-3 group). The group receiving 40 mg/kg reached similar values of healthy controls for ALT; whereas the same result occurred from the dose of 10 mg/kg for AST. The ideal dose, estimated from the mean of all metabolic parameters was approximately 30 mg/kg/day. Regarding the immunological profile, TNF-α significantly decreased in the BG groups compared to controls (p < 0.05), reaching better values than n-3 group and similar to healthy controls. No significant differences were found between the groups in IL-1β or IL-10 (p > 0.05). No histological changes were found in the pancreas, liver, or intestine due to treatment among diabetic animals.

**Conclusions:**

BG significantly reduced blood glucose as well as serum total cholesterol, LDL-c and TG. There was a hepatoprotective effect due to the reduction in ALT and AST and a reduction in TNF-α, indicating a modulation of the immune response. In general, BG effects were better than n-3 supplement (or at least comparable) depending on the dose.

## Introduction

Diabetes mellitus (DM) is a chronic disease characterized by the autoimmune or idiopathic destruction of pancreatic cells [1] and/or insulin resistance [2]. Diabetic individuals have several abnormalities in the metabolism of macronutrients [3], resulting in hyperglycemia and predisposition to the development of several comorbidities, such as atherosclerosis, arterial hypertension, stroke, and acute myocardial infarction [4]. These associated complications compromise the quality of life of the affected individuals, harming their emotional, physical, and social well-being [5]. Additionally, this disease puts a great financial burden to the health systems worldwide [6].

Conventional treatment of DM involves changes in lifestyle with an emphasis on food and nutrition education and regular physical activity [7], in addition sometimes to oral medicine [8] and/or insulin therapy [9]. Variations in blood glucose are a major challenge for patients with DM, especially type 1 DM [10]. Functional and nutraceutical foods have been investigated as adjuvants in the control of this disease [11]. In this context, prebiotics comprise substrates that are selectively utilized by host microorganisms conferring a health benefit. This broader definition includes even non-carbohydrate substances [12]. Fermentable soluble fibers modifyes intestinal microflora, promoting increase of *Lactobacillus* and *Bifidobacterium*, and decrease *Bacteroides* and *Clostridium* [13]. Besides, omega-3 poliunsatured free fatty acids (PUFA) supplementation decrease *Faecalibacterium*, and increase *Bacteroidetes* and butyrate-producing bacteria belonging to the *Lachnospiraceae* family [14, 15]. Evidence from systematic reviews evaluating randomized clinical trials indicate that probiotics and/or prebiotics (and symbiotics–combining both) present antidiabetic effects by interfering with the composition of the gut microbial environment, reducing intestinal endotoxin concentrations and decreasing energy absorption [16]. The consumption of up to 3 g/day of marine-based n-3 PUFAs is generally regarded as safe (GRAS) by US Food and Drug Administration (FDA) [15]. Yeast BG supplementation derived from *S. cerevisiae* has been approved for use in food supplements by the FDA and received GRAS status in 2008 (goverment revenue number [GRN]: 000239) at a maximum dose of 200 mg per serving [17], with the daily dose ranging from 100–500 mg [18].

Soluble fiber, including β-glucan (BG), has received attention due to its hypoglycemic [19–21] and hypocholesterolemic effects [22, 23], with consequent reduction in insulin resistance [24], hepatoprotection [19], and immunostimulation [25]. These effects can help decrease DM comorbidities [26] by forming a protective intestinal barrier, delaying the absorption of lipids and free cholesterol [27]. This barrier acts as one of the main defense mechanisms of the body and produces immunoregulatory signals [28].

The regular intake of fiber generates numerous health benefits [29]. However, the greater efficacy of BG depends on the time of consumption and the ingested dose [30]. Thus, studies that investigate the optimal intake dose are of public health interest to produce data that will enable cost savings, promoting the inclusion of fiber in the routine treatment of DM patients [31] and reducing the risk of toxicity in comparison to other treatments [32].

Most studies investigate the effects of cereal fibers. The present study aimed to evaluate the effects of the ingestion of different doses of BG (isolated from *Saccharomyces cerevisiae*), in comparison to n-3 supplement, on the metabolic and inflammatory profile of rats with streptozotocin-induced DM.

## Materials and methods

### Animals

The present study was approved by the Ethics Committee on Animal Use (CEUA) under protocol number 082/17. A total of 40 male rats of the *Wistar* breed (*Rattus norvegicus albinus)* were used. The animals were 11 weeks old, with a weight of 278.4 grams (± 19 g). These animals were subjected to quarantine (38 days) and acclimated for 7 days. Then the animals were randomly distributed into five groups (N = 7/group) and kept in collective boxes. One additional group was composed of healthy controls (n = 5). The animals were treated in a climate-controlled room with a constant temperature of 23 ± 2°C and a light-dark cycle of 12/12 h. Commercial food and water were provided *ad libitum* throughout the experiment.

### Experimental induction of diabetes

DM was induced by the intraperitoneal administration of 70 mg/kg of streptozotocin (STZ) (Sigma, ST. Louis, MO, USA) dissolved in ice-cold citrate buffer (pH 4.5) (4 °C) [33]. Induction was done at the end of the afternoon, and after 48 h, the animals were fasted for 8 h. Blood glucose was measured by incision of the tail tip with previous topic anesthesia by 1 mg/kg lidocaine ointment using the *Accu*-*Check Active* device (© 2016 *Roche Diabetes Care*, lot 06061982, Germany). Animals with blood glucose above 250 mg/dL were considered diabetic [34].

### Oral administration of β-glucan and fish oil

The doses of BG were given through gavage and diluted in 0.3 mL of saline solution daily (Table 1). The BG used was obtained from the extract of the cell wall of *Saccharomyces cerevisiae* [Macrogard (Açucareira Quatá S/A—Divisão Biorigin, Lençois Paulista, SP, Brazil; Composition: β-glucan—minimum 60.0%; raw protein—maximum. 8.0%; pH (2% solution) 4.0–7.0; ash—maximum 10.0 g/100 g].

**Table 1:** Doses of BG administered by gavage in animals with streptozotocin-induced DM (70 mg/kg)

Groups	Health Status	N^a^	Treatment^b^
Control	Healthy	5	Gavage Saline solution
n-3	Diabetic	7	Gavage 1000 mg/kg/day
0 mg	Diabetic	7	Gavage Saline solution
10 mg	Diabetic	7	Gavage 10 mg/kg/day of BG
20 mg	Diabetic	7	Gavage 20 mg/kg/day of BG
40 mg	Diabetic	7	Gavage 40 mg/kg/day of BG

^a^number of animals ^b^gavage

Commercially acquired fish oil capsules were broken and poured into an amber glass daily. The dose was also administered by oral gavage as listed in Table 1. According to the data reported by the manufacturer, the fish-derived oil used had 0.58 g eicosapentaenoic acid (EPA) and 0.37 g docosahexaenoic acid (DHA) for each 3 grams of the product.

### Collecting material for analysis

**Fig. 1:**
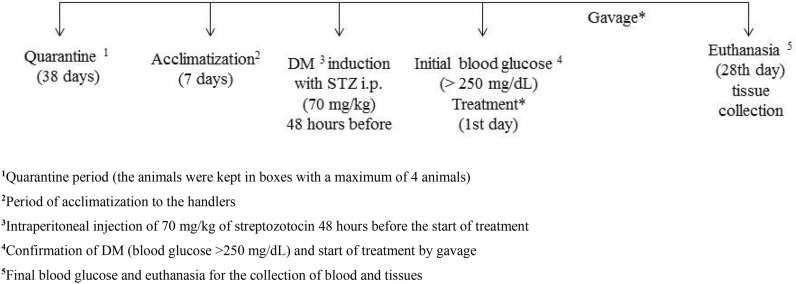
Experimental design over time

At the end of the 28-day experimental period, the animals were fasted for 8 h and euthanized through exsanguination by cardiac puncture  after anesthesia containing 50 mg/kg of sodium thiopental intraperitoneally. The blood samples were stored in sterile siliconized tubes (Vacuette^®^, Centerlab, Belo Horizonte, MG, Brazil), vacuum-sealed with clot activator (micronized silica particles), and plasma collection was performed using 4% EDTA (anticoagulant). Next, the tubes were centrifuged at 4000 rpm for 20 min. The liquid contents were poured into 2 mL Eppendorf tubes and stored in an ultrafreezer at − 80 °C until the time of analysis. Liver, small intestine, and pancreas were collected and kept in 10% formalin solution for 48 h (Fig. 1).

### Histopathological analysis of the liver, small intestine, and pancreas

After 48 h in 10% buffered formalin, the samples were conditioned in 70% ethanol. The fragments were processed in the Histotechnic (DM-70/12D OMA, São Paulo, SP, Brazil) and embedded in paraffin for cutting in a rotary microtome. The sections were cut to 4 µm thick. They were stained with hematoxylin and eosin for analysis of morphological features under light microscopy. Histological slides were evaluated by an experienced veterinary pathologist blind to the treatments. The pancreas were analyzed histologically for the number of cells present in the islets of Langerhans, and these were classified according to the following score: normal (−) (number of islet cells greater than 30) (+) mild lesion (number of islet cells 20–30); (++) moderate lesion (number of islet cells 10–20) (+++) severe lesion (number of islet cells less than 10) [35]. The liver and small intestine (duodenum, jejunum, and ileum) were analyzed histologically to identify the occurrence of lesions or any other type of microscopic alteration. Histological images were captured using coupled camera to a light microscope (CX31 binocular microscope, Olympus Optical do Brasil Ltda, São Paulo, SP, Brazil).

### Metabolic and immunological analyzes

The serum concentrations of triglycerides (TG), total cholesterol (TC), and HDLc were determined using a colorimetric assay according (Lab Test^®^, Lagoa Santa, Minas Gerais, Brazil). The level of LDLc was calculated using the Friedewald equation [36], where LDLc = TC − HDLc − TG/5. For these analyses, serum and reagent were pipetted together and then incubated in a water bath at 37 °C for 10 min. The reading was done at a wavelength of 505 nm for TG and TC at 500 nm for HDLc in an *Epoch Biotek* ^®^ spectrophotometer (Biotek^®^, Winooski, USA). Calculations were done with the formulas described below:

$${\text{TG and TC }}\left( {{\text{mg}}/{\text{dL}}} \right) = \frac{\text{Test Absorbance}}{\text{Standard Absorbance}} \times{\text{200}}$$

$${\text{HDL }}\left( {{\text{mg}}/{\text{dL}}} \right) \, = \frac{\text{Test Absorbance}}{\text{Standard Absorbance}} \times{\text{40}}$$

The liver enzymes aspartate aminotransferase (AST) and alanine aminotransferase (ALT) were obtained by plasma analysis in a colorimetric assay (Bioliquid^®^, Pinhais, Paraná, Brazil). The reagent (1 mL) was incubated for 3 min at 37 ℃. Next, the plasma samples were added, and the reading was taken at 340 nm after 1 min. The concentration was determined according to the following formula:

$${\text{Sample}}/{ \hbox{min} }\left( {{\text{U}}/{\text{L}}} \right) = \frac{{\delta \;{\text{Absorbance }}}}{\text{Total Absorbance }} \times {\text {1746}}$$

The serum concentrations of interleukin (IL)-1β, IL-10, and tumor necrosis factor alpha (TNF-α) were determined by enzyme-linked immunosorbent assay with commercial kits (Invitrogen^®^, Thermo Fisher Scientific, Vienna, Austria). Serological samples were diluted (1:5) and pipetted with the reagent. These were incubated for 120 min at room temperature (21°~ 25° C) in a 3-dimensional homogenizer KJMR-V^®^ (Global Equipment, *Global Trade Technology*, São Paulo, Brazil). The readings were taken at 450 nm in the *Epoch Biotek*^®^ spectrophotometer (Winooski, VT, USA).

### Statistical analysis

The data were compared by analysis of variance (ANOVA) followed by the Student–Newman–Keuls post hoc test (p < 0.05) in the statistical software Prism 5.0 (GraphPad Prism, CA, USA). Data are expressed as the mean ± standard deviation. For the regression model, the second-degree fit was performed using Excel software (Microsoft Excel, 2013).

## Results

**Fig. 2:**
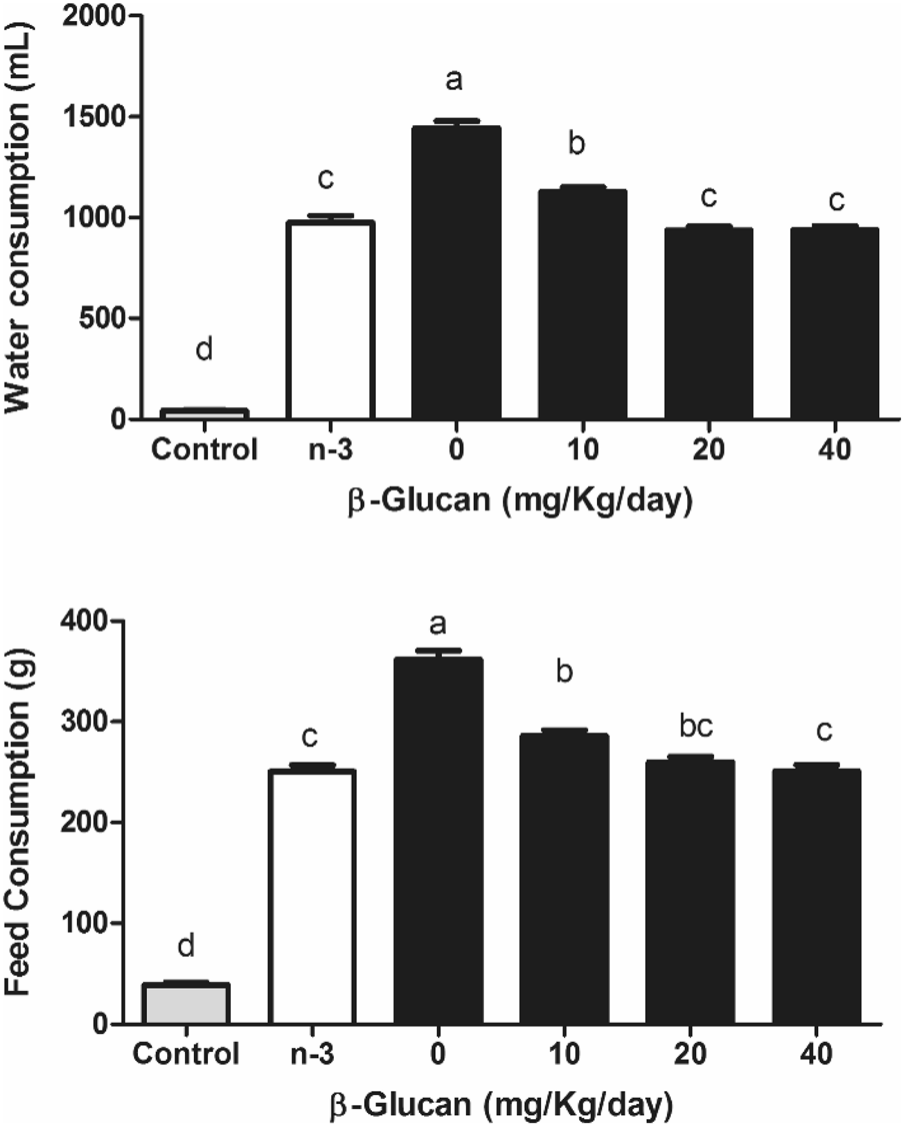
Daily water and food intake of diabetic Wistar rats induced by intraperitoneal injection of streptozotocin (70 mg/kg) and treated with different doses of β-glucan from *Saccharomyces cerevisiae* for 28 days. Different lowercase letters indicate significant differences by the Student–Newman–Keuls test at 5% probability (p < 0.05)

**Fig. 3:**
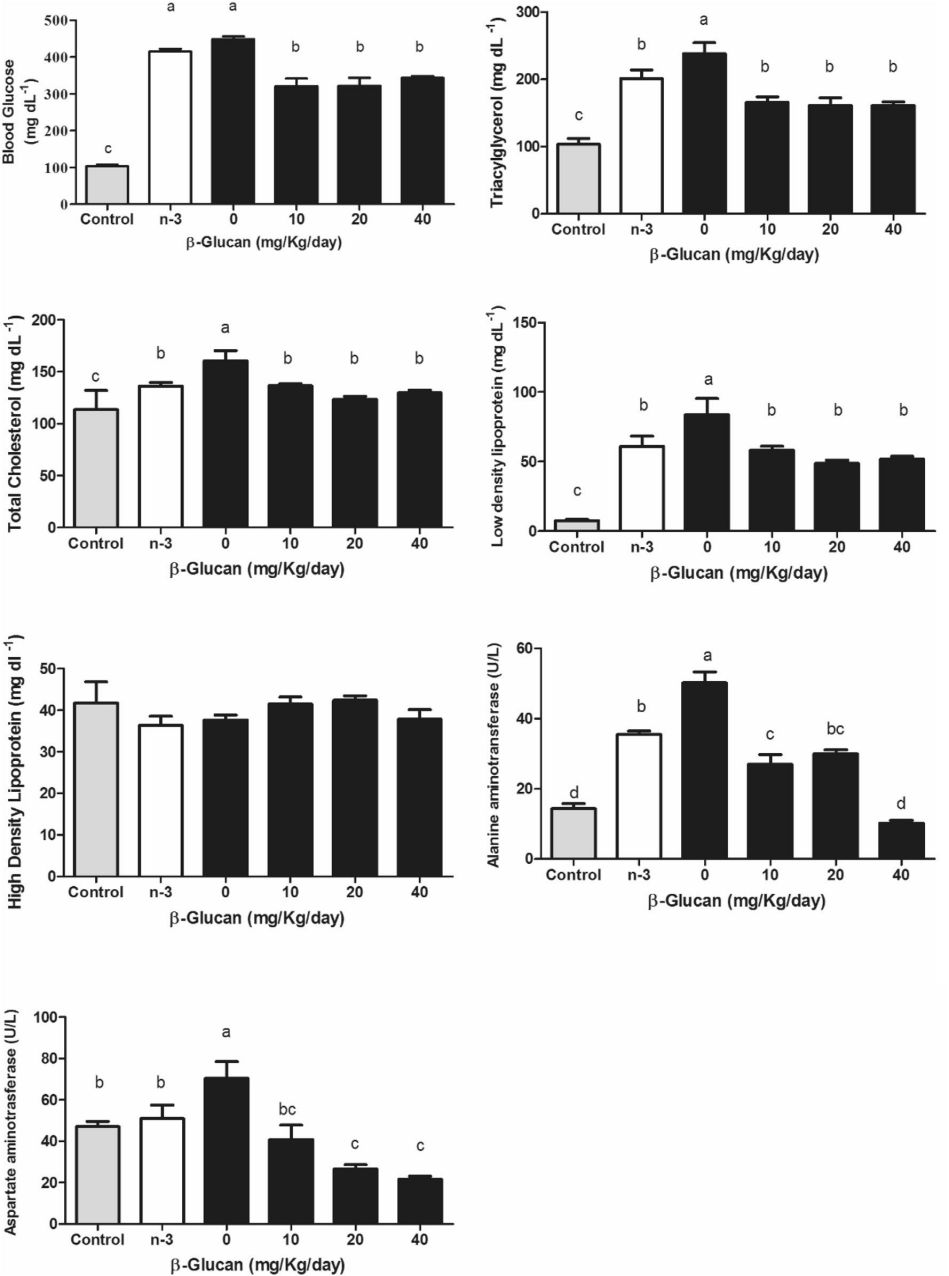
Metabolic parameters of diabetic Wistar rats induced by intraperitoneal injection of streptozotocin (70 mg/kg) and treated with different doses of β-glucan from *Saccharomyces cerevisiae* for 28 days. Different lowercase letters indicate significant differences by the Student–Newman–Keuls test at 5% probability (p < 0.05)

The water and food intake (Fig. 2) were higher in the vehicle (negative control group - 0 mg BG) and 10 mg/kg BG groups than the other treatments (p < 0.05), demonstrating the classic symptom of polydipsia and polyphagia, especially in the 0 mg group. However, consumption was still elevated in comparison to healthy controls (p < 0.05). There was a significant reduction in blood glucose (approximately 27%) at all nonzero BG doses (p < 0.05) compared with the 0 mg and omega-3 groups (Fig. 3). There was a significant reduction in total cholesterol (Fig. 3) above 10 mg/kg BG doses (approximately 23%), as well as in TG (Fig. 3) (32% reduction) and LDL-c (Fig. 3) (approximately 30%) (p < 0.05). On the other hand, no significant differences were seen in HDL-c (Fig. 3). In general, all BG presented similar results compared to omega-3 (p > 0.05), except for blood glucose levels in which BG presented better results (Fig. 3). Although several parameters significantly improved, the reached values were still higher than the healthy group. HDL-c was not different (p > 0.05) among all groups, including the healthy controls.

**Fig. 4:**
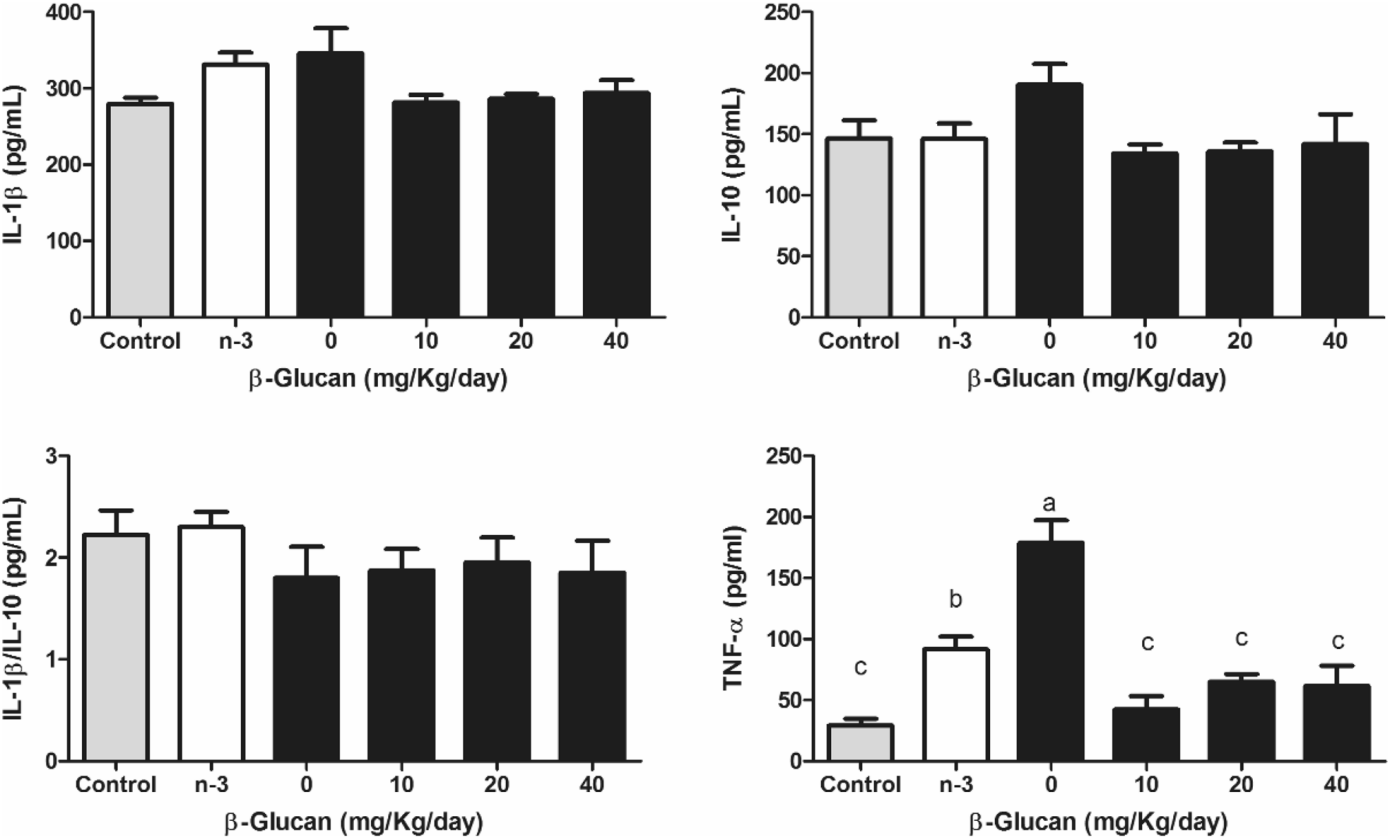
Inflammatory parameters of diabetic Wistar rats induced by intraperitoneal injection of streptozotocin (70 mg/kg) and treated with different doses of β-glucan from *Saccharomyces cerevisiae* for 28 days. Different lowercase letters indicate significant differences by the Student–Newman–Keuls test at 5% probability (p < 0.05)

Liver enzymes ALT and AST (Fig. 3) were both lower (up to 40% and 60%, respectively) in BG groups vs. the 0 mg group. There was a significant reduction in ALT in the omega-3 group, similar to the 20 mg BG group (p < 0.05). The group receiving 40 mg/kg reached similar values of healthy controls for ALT; whereas the same result occured from the dose of 10 mg/kg for AST. No significant differences were found between the groups in serum IL-1β, IL-10, or IL-1β/IL-10 ratio (Fig. 4b), but a significant reduction in TNF-α was observed when compared to 0 mg (p < 0.05), reaching healthy control values.

**Fig. 5:**
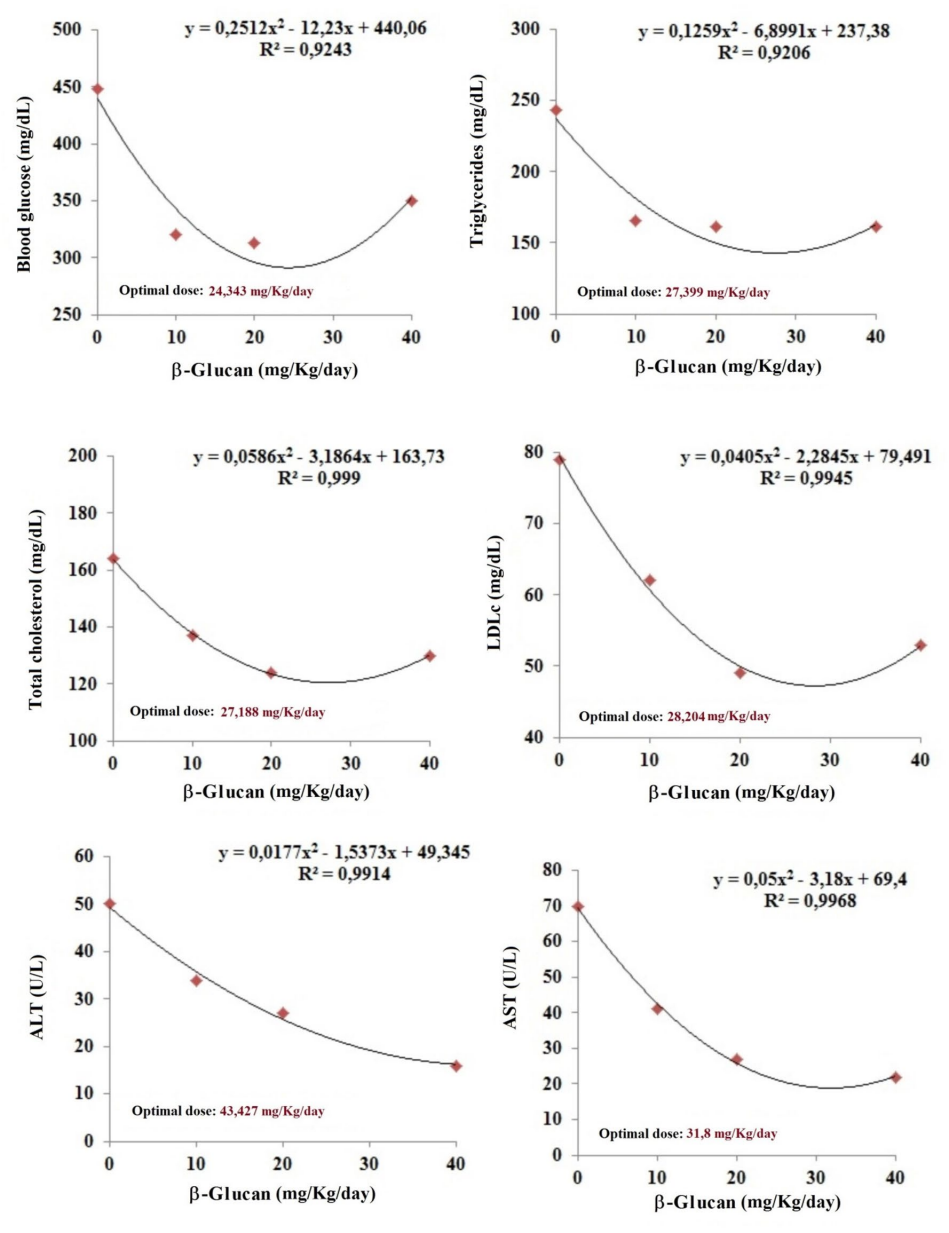
Adjustment of the second-order linear model according to the R^2^ coefficient of determination in diabetic Wistar rats induced by intraperitoneal injection of streptozotocin (70 mg/kg) and treated with different doses of β-glucan from *Saccharomyces cerevisiae* for 28 days

In order to estimate the best dose of BG for each parameter (blood glucose, TC, LDL-c, TG, ALT, and AST) were fit into a second-degree linear model (Fig. 5). The optimal dose was estimated by the mean of all parameters, and the value found was approximately 30 mg/kg/day. No significant changes were found in histological samples of the liver or duodenum, jejunum, or ileum submucosa, regardless of the treatment. According to the established criteria, there was a significant reduction in the number of pancreatic islet cells due to the induction of diabetes (Table 2), but no differences were found among treated or 0 mg groups (p > 0.05). Healthy controls present only normal scores, differing from all other diabetic groups.

**Table 2:** Histopathological scores of the pancreas of streptozotocin-induced diabetic rats (70 mg/kg) treated with different doses of yeast β-glucan

Treatment	Pancreatic islet cell number reduction score				
	Normal (−)	Mild (+)	Moderate (++)	Severe (+++)	
Control	5	0	0	0	+
n-3	0	3	3	1	*
0 mg	0	5	2	0	*
10 mg	0	4	2	1	*
20 mg	0	4	2	1	*
40 mg	0	4	3	0	*

*No statistically significant differences among diabetic groups according to the Kruskal-Wallis test (p > 0.05). + Significant difference in relation to diabetic groups according to the Kruskal-Wallis test (p < 0.05)

## Discussion

The present study demonstrated the beneficial effects of the consumption of BGs on blood glucose, reducing clinical signs of polyphagia and polydipsia. There were also positive effects on serum total cholesterol, LDL-c, and TG, in addition to a hepatoprotective effect. Water and food intake were significantly lower in the groups receiving BG regardless of the dose. This reduction may be associated with the ability of BG to promote satiety [37]. BGs increase central YY peptide secretion [38] and delay gastric emptying by increasing viscosity and water retention in the intestine [39] (reducing intestinal peristalsis, i.e., postprandial contractility is extended) aiding in glucose homeostasis [40] and reducing the appetite. The decrease in water and food intake with omega-3 supplementation also occurs due to its ability to modify the expression of neuropeptides related to appetite in the hypothalamic axis [41].

In the present study, the results of BG and n-3 were quite equivalent, with even better results for BG on reducing blood glucose levels depending on the dose. BG is able to reduce the absorption of glucose due to its ability to form a barrier (gel/viscose) in the intestine, causing a delay in the absorption of carbohydrates [42]. In addition, the capacity of yeast BG to aid in the decrease in glucose transporters SGLT1 and GLUT2 in the small intestine has been reported [43]. This same mechanism was also demonstrated for oat BG [44].

In obese and type 2 diabetes, the composition of the gut microbiota is associated to excessive ingestion of high-fat diets (HFD) and low-grade inflammation. Taking together, these factors play important roles for development of obesity and other chronic diseases [45]. Briefly, HFD generates microbiota shift increasing the expression of fat translocase, scavenger receptor CD36 and the scavenger receptor class B type 1 (SR-BI). SR-BI binds and incorporate lipids and lipopolysaccharides (LPS) to chilomicrons. After epithelial translocation, LPS is transferred to lipoproteins (such as HDL) and is directed to adipocytes. In this site, LPS contributes to M2 to M1 phenotype macrophage polarization and adipocyte hypertrophy [45]. Tryptophan-derived metabolites produced by the gut microbiota also controls the expression of the miR-181 family in white adipocytes that regulates energy expenditure and insulin sensitivity [46]. In the present study diabetes was induced by streptozotocin β-cell destruction, which approximates to type 1 diabetes. Even in this situation, we found positive effects of BG ingestion.

Microbial fermentation of prebiotics generates short-chain fatty acids (SCFA), such as acetate and butyrate, that have been shown to protect against oxidative and mitochondrial stress [47], enhance gut barrier, increase glucangon-like peptide 1 and 2 (GLP-1 and GLP-2) secretion [48], which delay gastric emptying and induce satiety [49]. Besides, incretins reduce hepatic expression of inflammatory and oxidative stress markers during obesity and diabetes [50]. GLP-1 stimulates insulin and reduces glucagon secretions from pancreatic α cells, reducing liver glucose output [51] and improving peripheral uptake of glucose [52]. Besides, SCFA inhibits lipolysis and reduces inflammation, enhancing energy metabolism regulation [16]. A recent study showed that SCFAs enhanced the viability of islets and β-cells, prevented STZ-induced cell apoptosis, viability reduction, mitochondrial dysfunction, and the overproduction of reactive oxygen species (ROS) and nitric oxide (NO) [47]. Mechanisms responsible for the efficacy of dietary n-3 PUFAs include reduction in IFN-γ, IL-17, IL-6, and TNF-α corroborating our findings (reduction in TNF-α) [53]. Indeed, n-3 PUFAs preventes lymphocyte infiltration into regenerating pancreatic islets, and elevates the expression of the β cell markers (Pdx1) and paired box 4 (Pax4) [53] what resembles our present findings on increased glucose control in animals ingesting n-3 supplement against control group.

Decreased glucose was also found in diabetic rats receiving doses of 6 mg and 12 mg of BG derived from fungus. The authors found significant reductions of 17% and 52% in blood glucose, respectively [54]. A significant reduction in blood glucose of 32% was also reported in a previous study of our group using a dose of 30 mg/kg BG for 28 days in streptozotocin-induced diabetic animals [19]. No significant differences were found in blood glucose levels with the use of omega-3 in the present study, corroborating previous studies in type 1 diabetic patients [55, 56]. Indeed, side effects of the use of omega-3  in glucose homeostasis have been reported, caused by increasing need for insulin [57], because polyunsaturated fatty acids can induce changes in the fluidity of cell membranes [58], decreasing the affinity of insulin for its receptors [57].

The biochemical parameters TC, TG, and LDLc also decreased significantly with the ingestion of BG. These hypoglycemic and hypolipidemic effects are also related to the ability of BG to form a gel barrier in the intestine, delaying the absorption of carbohydrates and lipids in enterocytes and consequently reducing cholesterol [59]. With the formation of the gel, there is an increase in the fecal bulk viscosity that prolongs gastric emptying [60], increasing the water layer with a consequent decrease in the uptake of cholesterol in the intestine [61] and greater elimination in the feces [62]. Among the main mechanisms of cholesterol reduction is the decreased absorption of bile salts due to the ability of BG to adsorb these salts, reducing their resorption and return to the liver [63]. Hepatic cholesterol reduction regulates the synthesis of the LDLc receptor. This fact generates increased uptake of the LDL-c by the liver, as well as negatively modulate the synthesis of 3′-hydroxy-3-methyl-glutaryl-coenzyme A reductase (HMG coA reductase), the enzyme responsible for cholesterol synthesis [63], through fermentation and production of SCFAs. Hypolipidemic effect of omega-3 was demonstrated in the present study. This effect seems to be related to increased EPA and DHA in the hepatic membrane [64]. In addition, it has been suggested that EPA can act as a second messenger, also reducingn HMG-coA reductase [65], the same effect attributed to BG. Shinozaki et al. [66] found that a dose of 1800 mg/day of EPA significantly reduced TC, TG, and LDLc after 6–24 months. Lobato et al. [19] demonstrated a 32% reduction in TG concentration and 41% reduction in ALT, demonstrating a hepatoprotective effect of BG. In addition, another study from our group showed a significant reduction in glucose, cholesterol, and TG levels in diabetic animals [59]. The reduction in blood glucose is directly related to the decrease in ALT due to the inhibition of the participation of this enzyme in the gluconeogenesis pathway [67].

In this study, BG did not affect HDLc. Previous meta-analysis was not able to determine whether dietary fiber intake was associated with HDLc metabolism [68]. However, it can be inferred that fiber intake reduced LDLc without reducing HDL-c, which is a benefit [26]. This process may have occurred due to the high level of cholesterol in the bloodstream (due to diabetes) and through processes triggered by epinephrine and hydrolysis enzymes, generating proportional synthesis of HDLc to carry free cholesterol to be metabolized in the liver [69]. It is important that HDLc was not decreased by BG because, given its association with reverse cholesterol transport, it can suppress the accumulation of cholesterol in peripheral tissues [70], also aiding in its systemic decrease. These results are consistent with previous research [71].

BG significantly reduced TNF-α blood levels in the present study, corroborating previous results [72] probably because of an increase in the intestinal barrier. These results were similar to omega-3 , in which EPA and DHA supplementation increases adiponectin and reduces TNF-α [73]. It is important to highlight the safety of BG for human and animal consumption. Yeast BG at different concentrations showed no adverse inflammatory, hematological or toxicological effects in mice [74]. Several studies report the safety of oral BGs consumption regardless of the source (oat, mushroom, or yeast) or used doses [75, 76].

Although we have some limitations regarding microbiota sequencing and lack of molecular markers quantification, the present study results may be speculated into potential translation into humans. A randomised, double-blind, placebo-controlled clinical trial testing BG from *Saccharomyces cerevisiae* was performed in overweight and obese subjects (3 g/day) for 12 weeks. Results indicated that daily supplementation was useful for improving body weight and waist circumference, without adverse effects [77]. Meta-analysis evaluating the effect of oat BG intake on glycaemic control of diabetic patients (using only randomized controlled trials) indicated that BG ingestion significantly lowered concentrations in fasting plasma glucose and glycosylated hemoglobin (HbA1c) [78]. Another meta-analysis also showed that beta-glucan extracted from oats were effective in decreasing fasting glucose and fasting insulin of T2D and tented to lower HbA1c [79]. Consistent with our results, another meta-analysis of clinical trials showed that β-glucan has a lowering effect on LDLc, non-HDLc and apoB [26]. Although oat and yeast β-glucans have some chemical differences, a previous study showed that as long as the purity of β-glucan is high, there is no difference among the sources of β-glucans [43, 80].

The broader definition of prebiotics including non-carbohydrate sources [12] opened space for other substances that could join this concept. Several substances such as Polydextrose (PDX), Xylo-oligosaccharides (XOS), Pectic-oligosaccharides (POS), Gluco-oligosaccharides, Malto-oligosaccharides, Isomaltooligosaccharides (IMO), Soya-oligosaccharides (SOS), Fenugreek, Gold-based nanomaterials, selenium compounds, and nanoceria can be considered [13, 81] candidates. In this sense, even omega-3 can be considered a candidate if we consider this definition.

Our results highlight the improvement of important metabolic parameters such as blood glucose levels and liprotein profile in a dose-dependent manner after consuming yeast BG. These outcomes add some new information regarding potential preventive and therapeutic care for type 1 diabetes, which often presents difficulties in control clinical set. The future of prebiotic research will probably include more studies focusing on the specificity of prebiotics for intestinal bacteria [13]. Clinical trials investigating the role of yeast BG in both type 1 and type 2 diabetic patients are necessary in order to establish optimal clinical protocols for general care. Dietary fiber ingestion of 100–500 mg/day has been reported as safe [18]. BGs may be consumed in different formulations; such as breakfast cereals or baked goods [22].

## Conclusion

The consumption of *Saccharomyces cerevisiae* BGs demonstrated promising effects in the treatment of DM by decreasing metabolic parameters (blood glucose, total cholesterol, LDLc, and TG) in addition to its hepatoprotective effect through the reduction in ALT and AST. It significantly reduced blood levels of TNF-α. The optimal estimated dose for the observed benefits in diabetic rats was around 30 mg/kg/day. In general, BG effects were better than n-3 supplement (or at least comparable) depending on the dose.

## Data Availability

The datasets used and/or analysed during the current study are available from the corresponding author on reasonable request.
